# Myopia Progression in School-Age Children During the COVID-19 Pandemic

**DOI:** 10.3390/jcm13226849

**Published:** 2024-11-14

**Authors:** Gülce Gökgöz Özışık, Hayati Yilmaz

**Affiliations:** 1 Department of Ophthalmology, Faculty of Medicine, Hitit University, 19030 Corum, Turkey; 2 Department of Ophthalmology, Faculty of Medicine, Maltepe University, 34844 Istanbul, Turkey; hayati.yilmaz@maltepe.edu.tr

**Keywords:** COVID-19, myopia, myopia progression, school-age children, pandemic

## Abstract

**Objectives:** This study aimed to investigate changes in refraction error in myopic school-age children during the COVID-19 pandemic. **Methods**: The data of 825 myopic children aged 7–18 years were retrospectively screened from the hospital data access system. The cycloplegic prescriptions of the patients in 2018, 2019, 2020, and 2021 were recorded. The patients were divided into three groups according to their ages: ≤10 years (Group A), 11–14 years (Group B), and ≥15 years (Group C). The mean refraction values and annual progression values were compared between the years and age groups. **Results**: The mean age of the patients was 13.8 ± 3.17 years. Statistical analysis for the overall sample indicated that the annual myopia progression significantly differed between 2018 and 2021 (−0.42 ± 0.37 and −0.53 ± 0.47, respectively) (*p* < 0.001), and there was also a significant difference in myopia progression observed in all years in the younger age group (−0.34 ± 0.44 for 2018, −0.50 ± 0.49 for 2019, and −0.76 ± 0.59 for 2020). The highest progression (−0.76 ± 0.59) was determined in the younger age group in 2020. Linear regression analysis showed a negative correlation between myopia progression from 2020 to 2021 and age (B = 0.049 and *p* < 0.001). **Conclusions**: Myopia progression has increased in school-age children during COVID-19, with the younger age group being more affected. During the COVID-19 pandemic, myopia progression in younger children has increased statistically significantly. Thus, at times when distance learning is required, it would be appropriate to plan by taking into account the myopia progression of children.

## 1. Introduction

Myopia is a refractive error in which distant vision is impaired. Although the incidence of myopia is increasing worldwide, It is even more common in Far East countries and is called an “epidemic of myopia” in this region. [1,2]. In a meta-analysis, it was suggested that half of the world’s population would be myopic by 2050, and 10% of these cases would be severely myopic [3]. Advanced myopia may be associated with vision-threatening pathologies, such as retinal detachment, choroidal neovascularization, cataracts, and glaucoma at advanced ages [4].

Both genetic and environmental factors play a role in the etiology of myopia. However, it has been suggested that genetic factors cannot explain the rapid progress of this condition in a short time. Among environmental factors, near work and spending less time outside have been particularly emphasized in the progression of myopia [2,5].

According to the United Nations Educational, Scientific and Cultural Organization data, governments have suspended face-to-face education in many countries due to the COVID-19 pandemic [6]. It has been stressed that the closure of schools has had unfavorable effects on children’s physical and mental health [7]. Recent publications have discussed the impact of quarantine implementations on myopia progression [8,9,10].

In Turkey, the first COVID-19 case was reported on 11 March 2020, and the distance education system was started for all age groups on 12 March 2020. During this period, students continued their education at home with long-term exposure to screens. Face-to-face education was started to be implemented two days a week on 28 September 2020 for Grade 1 students and on 12 October 2020 for those aged 6, 7, 8, 9, 13, and 17 years. Although there were also intermittent lockdown measures during the remainder of the 2020–2021 academic year, education continued as mentioned above until 21 June 2021 [11].

This study aimed to detect and discuss changes in refraction in school-age children with myopia during the COVID-19 pandemic.

## 2. Materials and Methods

### 2.1. Demographic Data

In this retrospective, cross-sectional study, the files of children aged 7–18 years were retrospectively screened from the hospital data management system (AKGÜN web HBYS, Ankara, Türkiye). Patients who were presented to the outpatient clinics of the Hitit University Erol Olcok Training and Research Hospital, Department of Ophthalmology between August 2020 and August 2021 and had been diagnosed with myopia before 2020 were included in this study. The study data covered the period September 2018–August 2021. We included the right eye of the patients if both eyes had myopia or the eye that had myopia. Excluded from the study were patients with the diagnoses of any other ocular pathologies, such as cataract or strabismus; those with a refractive power greater than −0.50 diopter (D) (emmetropia and hyperopia); those who had received any preventive treatment for myopia progression, including wearing bifocal/progressive eyeglasses or orthokeratology lenses and medical therapy; and those who required only one prescription for eyeglasses or did not require any prescription for eyeglasses during the COVID-19 pandemic period. The age of children in this study refers to their age on the last examination date. All the patients had recorded complete ophthalmological examination data, including the best-corrected visual acuity measurement, slit-lamp biomicroscopy, and dilated fundoscopy.

This research was reviewed by an independent ethical review board and conforms with the principles and applicable guidelines for the protection of human subjects in biomedical research. This study was carried out under the tenets of the Declaration of Helsinki.

### 2.2. Refractive Power and Progression Calculations

For all the patients, autorefractometry data were obtained after 45 min following the instillation of two drops of cyclopentolate hydrochloride 1% (Sikloplejin eye drops, Abdi Ibrahim Ilac, İstanbul, Turkey) at an interval of 10 min. The refractive data of the pre-pandemic and pandemic periods were screened, and at least two and at most four refractive powers measured during the pre-pandemic period (2018 and/or 2019) and the pandemic period (2020 and/or 2021) were recorded in spherical equivalent (SE). The differences between the refractive powers of 2018 and 2019 (progression from 2018 to 2019), 2019 and 2020 (progression from 2019 to 2020), and 2020 and 2021 (progression from 2020 to 2021) were simply calculated with the subtraction of each refractive power from the refractive power of the following after. Then, for statistical analyses, the patients were divided into three groups according to their age: ≤10 years (Group A), 11–14 years (Group B), and ≥15 years (Group C).

### 2.3. Statistical Analyses

Jamovi ver. 1.6 (computer software, https://www.jamovi.org, accessed on 3 March 2022) was used for statistical analyses. Quantitative variables were defined as mean and standard deviation and qualitative variables as percentages. The Shapiro–Wilk test was used to evaluate whether the sample came from a normally distributed population. To compare the SE values between each year, parametric repeated-measure analysis of variance (ANOVA) and post hoc Tukey or non-parametric Friedman’s repeated-measure ANOVA test was used, and the post hoc Durbin–Conover test was conducted for pairwise comparisons. One-way ANOVA or the Kruskal–Wallis test was used to compare the mean SE obtained from each year between the age groups. The annual progressions were compared using a parametric paired *t*-test or non-parametric Wilcoxon rank test. Lastly, the overall effects of age and gender on annual progressions were calculated independently of each other using the linear regression analysis, with age as a covariate and gender as a factor for each year’s progression. A *p*-value of less than 0.05 was considered statistically significant.

## 3. Results

### 3.1. Demographic Data Results

Of the 3794 patients screened, 1283 had been diagnosed with myopia, and 825 met the inclusion criteria. The number of female patients was 541 (65.6%). The mean age of the patients was 13.8 ± 3.17. Concerning the age groups, 144 patients were ≤10 years old, 314 were 11–14 years, and 367 were ≥15 years. The number of cases with eyeglasses prescription data was 499 for 2018, 681 for 2019, 445 for 2020, and 741 for 2021. Only 205 patients had the SE data for all four years.

### 3.2. Refractive Power and Progression Data

Table 1 presents the demographic data of the patients and the mean SE obtained from each year. The mean refractive power of the prescribed eyeglasses (mean SE) in 2019 and 2020 significantly differed between the age groups (*p* < 0.001 for both, Kruskal–Wallis test). For the 2019 mean SE values, the pairwise comparisons revealed significant differences between Group A and Group B, Group A and Group C (*p* = 0.011 and *p* = 0.003, respectively); however, there was no significant difference between Group B and Group C (*p* = 0.893). The pairwise comparisons between the age groups regarding the mean 2020 SE values were similar to the 2019 results (*p* = 0.004 for Group A vs. Group B, 0.005 for Group A vs. Group C, and 0.950 for Group B vs. Group C).

No significant difference was observed for the 2018 and 2021 mean SE values between the age groups (*p* = 0.135 and 0.074, respectively, Kruskal–Wallis test). Friedman’s repeated-measure ANOVA and post hoc Durbin–Conover tests revealed significant differences between the mean SE values of each year for the overall sample and for all age groups (*p* < 0.001 for all).

**Figure 1:**
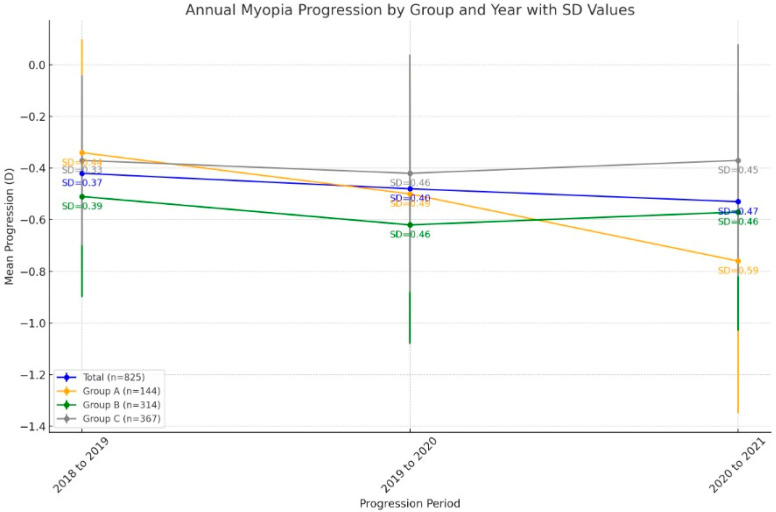
This graph illustrates the annual myopia progression from 2018 to 2021 across four age groups: patients aged ≤ 10 (Group A), 11–14 years (Group B), and ≥15 years (Group C), and the total sample. The data show that the youngest group (Group A) experienced a faster progression rate, especially during the 2020–2021 period. The SD (standard deviation) values, shown next to each point, reflect the variability in progression.

Annual myopia progression data are given in Table 2. For the overall sample, there was a significant difference in the progression rates of SE between 2018 and 2020 (*p* < 0.001, Wilcoxon rank test). However, the progression rates of SE did not significantly differ between 2018 and 2019 or between 2019 and 2020 (*p* = 0.065 and 0.861, respectively, Wilcoxon rank test). The mean myopia progression rates in 2018, 2019, and 2020 all significantly differed from each other in Group A [*p* = 0.001 (progression from 2018 to 2019 vs. from 2019 to 2020); 0.018 (progression from 2019 to 2020 vs. from 2020 to 2021); and <0.001 (progression from 2018 to 2019 vs. from 2020 to 2021); Wilcoxon rank test]. However, there was no significant difference between the progression rates of different years in Group B or Group C (*p* > 0.05 for all, Wilcoxon rank test, Table 2). Figure 1 shows the change in myopia according to age groups by years.

The linear regression models revealed that only the progression from 2020 to 2021 was significantly associated with age (r = 0.318 and *p* < 0.001, Table 3). The progression from 2020 to 2021 was found to be decreased with increasing age (negative correlation) (B = 0.049). The progression rates were not associated with gender in any of the evaluation years (*p* > 0.05 for all, Table 3).

## 4. Discussion

This study evaluated cycloplegic refraction values and myopia progression before and during the COVID-19 pandemic in myopic children aged 7–18 years. We also divided the patients into groups (Groups A to C) according to their ages. The mean refraction values for both the overall sample and each group showed a significant decrease over the years. The annual progression of myopia between 2018 and 2021 exhibited significant differences in the younger age group. The linear regression analysis revealed a negative correlation between myopia progression from 2020 to 2021 and age.

Many studies have investigated the relationship between myopia progression and exposure to bright light, near work, circadian rhythm, vitamin D level, light spectrum, physical activity, and peripheral defocus. According to the current data, the most evident finding concerning this condition is that spending time outside reduces the progression of myopia. Exposure to bright light and peripheral defocus are the strongest mechanisms of this effect. The effect of light brightness on myopia progression has been demonstrated in animal and human experiments. Objects out of sight generally give the eye a uniform diopter in the outside environment, resulting in minimal external defocus for the peripheral retina. Although the effect of near work and accommodation on myopia progression remains controversial, it has undeniable importance. Another factor in this regard is artificial light. Artificial light and sunlight contain a different spectrum of lights at different proportions. Since each wavelength has different diffraction, artificial light, which is relatively poor in blue, green, and ultraviolet light, creates a defocus on the retina, triggering eye enlargement and myopia progression. Spending time outside is the most substantial factor that reduces myopia progression, although its exact mechanism has not yet been clarified [2,5,12].

In Turkey, schools were closed for most of 2020 [6]. Publications suggest that time spent at home may have increased myopia progression during this period [8,9,10].

In a study conducted before the COVID-19 pandemic to prevent myopia progression in Far East countries in the 6–12-year age group, the annual change in refractive error was determined as −0.84 D in the placebo group [13]. In a large-scale study undertaken in China, the yearly refraction change was reported to be −0.35 D in school-age children with myopia [14]. In another study conducted in the West, the annual progression of myopia in myopic children was reported as −0.34 D [15]. In India, the annual myopia progression was −0.27 D in children aged 5–15 years [16]. In 2012, a meta-analysis reported that Asian children had higher myopia prevalence and myopia progression than European children. In that meta-analysis, the annual myopia progression was reported as −0.82 D in Asian children and −0.55 D in European children [17]. 

According to a study conducted in Turkey before the pandemic, the annual myopia progression was −0.30 D in children aged 9–14 years. It was correlated with the daily time spent on reading and writing [18]. Our study found the annual myopia progression during the pre-pandemic period (2018–2019) to be −0.42 D for the 7–18-year group. Similarly, the annual myopia progression in this group was −0.48 D for 2019–2020, while it reached −0.53 D for 2020–2021. When all age groups were evaluated, the rate of myopia increased over each year in our study, but the difference between the 2018 and 2020 rates was statistically significant. The data showed that the main group that created this difference was the young age group. In the younger age group of our research, the progression from 2020 to 2021 was found to be −0.76 D, which is close to the annual progression value obtained from the Asian study [17]. Studies have emphasized that myopia is more severe in younger children during the COVID-19 period and that these children are more affected by environmental factors [9,19].

In a study during the COVID-19 pandemic, Wang et al. found a significant difference in non-cycloplegic refraction values between 2019 and 2020 [20]. In our study, the mean annual refractive error significantly decreased over the years in all myopic children aged 7–18 years and in each age group.

A study from China compared school screenings conducted in 2015, 2019, and 2020. Compared to the previous years, a significant myopia tendency was detected in the 6–13-year group in the 2020 screening. The authors emphasized that differences in refractive errors between the years were not as significant in previous screenings as in the 2020 screening. There was an increase in the prevalence of myopia in the 6–8-year group in the 2020 screening. The authors also stated that the refraction error in 2020 was higher in the younger age group than in previous years. The change in refractive error during the 2020 screening was attributed to the implemented lockdown measures [9]. Similarly, myopia progression was significantly higher in the younger age group in our study, but different from the previous study, we evaluated cycloplegic refractive errors in our sample. Furthermore, the research mentioned above was a screening study that included all types of refractive errors, distinguishing them from our work.

Ma et al. evaluated children aged 8–10 years in terms of changes in cycloplegic SE over two seven-month periods; one before the pandemic and the other following lockdown measures implemented due to the COVID-19 pandemic. The seven-month SE change during the lockdown period (−0.93 D) was more significant compared to both the control group (−0.36 D) and pre-pandemic evaluation (−0.33 D) [21]. However, it should be considered that the sample of that study included a mixed patient group of myopia, hyperopia, and emmetropia of a narrow age range. In our study, only myopic patients were included in the study.

Ma et al. emphasized that the change in SE during the pandemic was more significant compared to the pre-pandemic period in myopic children aged 7–12 years. This progression was suggested to be significantly correlated with increasing screen use time. Advanced age was shown to protect against myopia progression [22]. In our study, the younger age group aged 7–10 years had the highest myopia progression in the inter-group comparisons and comparison of pre-pandemic and pandemic periods. In addition, our linear regression model revealed a significant negative correlation between myopia progression from 2020 to 2021 and age. Two hypotheses have been put forward for this sensitivity of the younger age group to myopia progression: this age group may have been more affected by lifestyle changes due to lockdown measures and may have spent more time at home, or they may be more sensitive to the effect of lockdown on myopia during the pandemic period [23]. In a study conducted during the COVID-19 pandemic, Xu et al. reported that the younger student group was more sensitive to myopia. Online time in this student group increased to a much greater extent than in the older age group. It was emphasized that the increase in myopia prevalence and myopia progression was correlated with students’ online time [24]. 

In a study conducted in Turkey during the COVID-19 period, cycloplegic refractive error was evaluated in children aged 8–17 years. The annual myopia progression was found to be −0.71 D for 2019–2020, and it was emphasized that this value significantly differed compared to the previous years. The authors emphasized the effect of the participants’ housing type and outdoor activity time on myopia progression [25]. This study can be compared with our results in the sample’s similar age and the cycloplegic refractive error values. We found the annual myopia progression to be −0.48 D for 2019–2020 among 289 children. Unlike the previous study, we also grouped our participants according to age groups and determined the progression value to be −0.76 D in the younger age group for 2020–2021.

We did not evaluate the patients participating in our study’s screen time, physical activity, and outdoor time parameters. Still, many publications have associated myopia progression during the COVID-19 period with these parameters [20,21,25,26,27].

It is clear that time spent at home has increased during the COVID-19 lockdown measures. In this period, screen use may have increased in children who have been deprived of both social and physical activities. Distance education constitutes an essential part of screen usage time. Many mechanisms of myopia progression discussed to date have become even more active during the lockdown measures. In a survey conducted with the parents of children aged 6–13 years in Turkey, 71.7% of the parents stated that their children’s screen use time increased during the lockdown, and the daily screen use time was determined as 6.42 h per day during this period [28]. For screen use time, the American Academy of Pediatrics recommends under 1 h per day for children aged 2–5 years and a consistent limit for children over six years [27]. 

Our research has certain limitations. First, data were collected retrospectively. Second, other parameters that can affect myopia progression, such as screen time, physical activity, and outdoor time, were not evaluated. The third limitation is that axial lengths could not be obtained due to the retrospective nature of our study. On the other hand, the strengths of our study are the annual progression data obtained from the records of the same individuals, the high number of patients in the sample, and the evaluation of cycloplegic refractive values.

To our knowledge, this is the first study to compare cycloplegic refraction values and myopia progression between pre-pandemic and pandemic periods according to different pediatric age groups.

## 5. Conclusions

The COVID-19 pandemic has had many adverse physical and psychological effects on children. Due to the unpredictable course of the pandemic, similar lockdown measures may be necessary again both during the current pandemic and in other possible future pandemics or natural disasters. In such periods, it is essential to organize the distance education process, considering the time spent by the children studying closely, the time they use screened devices, and the time spent in the open area.

## Figures and Tables

**Table 1:** Demographic and clinical findings of the study groups.

	Total (*n* = 825)		Group A (*n* = 144)		Group B (*n* = 314)		Group C (*n* = 367)		*p*
Mean age (years) (SD) [range]	13.8 (3.17) [7,8,9,10,11,12,13,14,15,16,17,18]		8.60 (1.33) [7,8,9,10]		12.9 (1.11) [11,12,13,14]		16.6 (1.20) [15,16,17,18]		<0.001 ^a^
female (n) [%]	541 [5.6]		89 [61.8]		208 [66.2]		235 [64.0]		0.113 ^b^
	n	mean (D, SD)	N	mean (D, SD)	n	mean (D, SD)	N	mean (D, SD)	
SE_2018	499	−1.77 (1.42)	84	−1.49 (1.36)	178	−1.84 (1.52)	237	−1.81 (1.36)	0.135 ^c^
SE_2019	681	−1.93 (1.50)	114	−1.50 (1.29)	268	−1.99 (1.56)	299	−2.05 (1.49	<0.001 ^c^
SE_2020	445	−2,49 (1.83)	106	−1.95 (1.42)	148	−2.70 (2.00)	191	−2.64 (1.83)	<0.001 ^c^
SE_2021	741	−2.77 (1.76)	132	−2.47 (1.65)	292	−2.81 (1.80)	317	−2.86 (1.76)	0.074 ^c^
*p*	<0.001 ^d^		<0.001 ^d^		<0.001 ^d^		<0.001 ^d^		

Group A; patients ≤10 years old, Group B; patients 11 to 14 years old, Group C; patients ≥15 years old. SD; standard deviation, D; diopter, a; one-way ANOVA test results, b; chi-square test results, c; Kruskal–Wallis test results (comparison of the mean SE of each year among age groups), d; Friedman’s repeated-measure ANOVA test results (comparison of the mean SE of each year from each other).

**Table 2:** Comparison of annual myopia progression in pre-pandemic and pandemic years.

		Mean (D, SD)	*n* ^a^	*n* ^b^	*n* ^c^	P^a^	P^b^	P^c^
Total (n = 825)	progression from 2018 to 2019	−0.42 (0.37)	237	289	205	0.065	0.861	**<0.001**
	progression from 2019 to 2020	−0.48 (0.40)						
	progression from 2020 to 2021	−0.53 (0.47)						
Group A (n = 144)	progression from 2018 to 2019	−0.34 (0.44)	60	74	60	**0.001**	**0.018**	**<0.001**
	progression from 2019 to 2020	−0.50 (0.49)						
	progression from 2020 to 2021	−0.76 (0.59)						
Group B (n = 314)	progression from 2018 to 2019	−0.51 (0.39)	73	104	66	0.249	0.850	0.502
	progression from 2019 to 2020	−0.62 (0.46)						
	progression from 2020 to 2021	−0.57 (0.46)						
Group C (n = 367)	progression from 2018 to 2019	−0.37 (0.33)	103	111	79	0.400	0.059	0.499
	progression from 2019 to 2020	−0.42 (0.46)						
	progression from 2020 to 2021	−0.37 (0.45)						

Group A; patients ≤10 years old, Group B; patients 11 to 14 years old, Group C; patients ≥15 years old. P^a^, P^b^, and P^c^: Wilcoxon rank test results of the comparison of mean myopia progression between 2018 with 2019, 2019 with 2020, and 2018 with 2020, respectively. n^a^, n^b^, and n^c^: number of paired myopia progression calculations of 2018 with 2019, 2019 with 2020, and 2018 with 2020. *p* < 0.05 means statistical difference.

**Table 3:** Associations of the myopia progression rates with age and gender in the pre-pandemic and pandemic years.

		R	B	95% CI		*p*
Progression from 2018 to 2019	Age	0.143	0.009	−0.003	0.021	0.145
	Gender		−0.027	−0.186	0.095	0.198
Progression from 2019 to 2020	Age	0.033	0.000	−0.015	0.013	0.897
	Gender		0.032	−0.070	0.135	0.536
Progression from 2020 to 2021	Age	0.318	0.049	0.034	0.065	**<0.001**
	Gender		−0.026	−0.135	0.082	0.628

R; regression model fit measurement, B; regression coefficient (estimate), CI; confidence interval, *p*; results of the linear regression models. *p* < 0.05 means statistical difference.

## Data Availability

The raw data supporting the conclusions of this article will be made available by the authors upon request.

## References

[1] Bourne R.R., Stevens G.A., White R.A., Smith J.L., Flaxman S.R., Price H., Jonas J.B., Keeffe J., Leasher J., Naidoo K. (2013). Vision Loss Expert Group. Causes of vision loss worldwide, 1990–2010: A systematic analysis. Lancet Glob. Health.

[2] Dolgin E. (2015). The myopia boom. Nature.

[3] Holden B.A., Fricke T.R., Wilson D.A., Jong M., Naidoo K.S., Sankaridurg P., Wong T.Y., Naduvilath T.J., Resnikoff S. (2016). Global Prevalence of Myopia and High Myopia and Temporal Trends from 2000 through 2050. Ophthalmology.

[4] Jones D., Luensmann D. (2012). The prevalence and impact of high myopia. Eye Contact Lens.

[5] Lingham G., Mackey D.A., Lucas R., Yazar S. (2020). How does spending time outdoors protect against myopia? A review. Br. J. Ophthalmol..

[6] UNESCO (2021). Global Monitoring of School Closures Caused by COVID-19. https://en.unesco.org/covid19/educationresponse/.

[7] Wang G., Zhang Y., Zhao J., Zhang J., Jiang F. (2020). Mitigate the effects of home confinement on children during the COVID-19 outbreak. Lancet.

[8] Pellegrini M., Bernabei F., Scorcia V., Giannaccare G. (2020). May home confinement during the COVID-19 outbreak worsen the global burden of myopia?. Graefes Arch. Clin. Exp. Ophthalmol..

[9] Wang J., Li Y., Much D.C., Wei N., Qi X., Ding G., Li X., Li J., Song L., Zhang Y. (2021). Progression of Myopia in School-Aged Children After COVID-19 Home Confinement. JAMA Ophthalmol..

[10] Klaver C.C.W., Polling J.R., Enthoven C.A. (2021). 2020 as the Year of Quarantine Myopia. JAMA Ophthalmol..

[11] Republic of Turkey Ministry of National Education Yuz Yuze Egitimde Ikinci Asama Basladi, Milyonlarca Ogrenci Okullariyla Bulustu. Updated 12 October 2020. https://www.meb.gov.tr/yuz-yuze-egitimde-ikinci-asama-basladi-milyonlarca-ogrenci-okullariyla-bulustu/haber/21787/tr.

[12] Rose K.A., Morgan I.G., Ip J., Kifley A., Huynh S., Smith W., Mitchell P. (2008). Outdoor activity reduces the prevalence of myopia in children. Ophthalmology.

[13] Tan D.T., Lam D.S., Chua W.H., Shu-Ping D.F., Crockett R.S., Asian Pirenzepine Study Group (2005). One-year multicenter, double-masked, placebo-controlled, parallel safety and efficacy study of 2% pirenzepine ophthalmic gel in children with myopia. Ophthalmology.

[14] Zhao J., Mao J., Luo R., Li F., Munoz S.R., Ellwein L.B. (2002). The progression of refractive error in school-age children: Shunyi district, China. Am. J. Ophthalmol..

[15] Grosvenor T., Perrigin D.M., Perrigin J., Maslovitz B. (1987). Houston Myopia Control Study: A randomized clinical trial. Part II. Final report by the patient care team. Am. J. Optom. Physiol. Opt..

[16] Saxena R., Vashist P., Tandon R., Pandey R.M., Bhardawaj A., Gupta V., Menon V. (2017). Incidence and progression of myopia and associated factors in urban school children in Delhi: The North India Myopia Study (NIM Study). PLoS ONE.

[17] Donovan L., Sankaridurg P., Ho A., Naduvilath T., Smith E.L., Holden B.A. (2012). Myopia progression rates in urban children wearing single-vision spectacles. Optom. Vis. Sci..

[18] Öner V., Bulut A., Oruç Y., Özgür G. (2016). Influence of indoor and outdoor activities on progression of myopia during puberty. Int. Ophthalmol..

[19] Zhang X.J., Zhang Y., Kam K.W., Tang F., Li Y., Ng M.P.H., Young A.L., Ip P., Tham C.C., Chen L.J. (2023). Prevalence of Myopia in Children Before, During, and After COVID-19 Restrictions in Hong Kong. JAMA Netw. Open.

[20] Wang W., Zhu L., Zheng S., Ji Y., Xiang Y., Lv B., Xiong L., Li Z., Yi S., Huang H. (2021). Survey on the Progression of Myopia in Children and Adolescents in Chongqing During COVID-19 Pandemic. Front. Public Health.

[21] Ma D., Wei S., Li S.M., Yang X., Cao K., Hu J., Fan S., Zhang L., Wang N. (2021). Progression of myopia in a natural cohort of Chinese children during COVID-19 pandemic. Graefes Arch. Clin. Exp. Ophthalmol..

[22] Ma M., Xiong S., Zhao S., Zheng Z., Sun T., Li C. (2021). COVID-19 Home Quarantine Accelerated the Progression of Myopia in Children Aged 7 to 12 Years in China. Investig. Ophthalmol. Vis. Sci..

[23] Chang P., Zhang B., Lin L., Chen R., Chen S., Zhao Y., Qu J. (2021). Comparison of Myopic Progression before, during, and after COVID-19 Lockdown. Ophthalmology.

[24] Xu L., Ma Y., Yuan J., Zhang Y., Wang H., Zhang G., Tu C., Lu X., Li J., Xiong Y. (2021). COVID-19 Quarantine Reveals That Behavioral Changes Have an Effect on Myopia Progression. Ophthalmology.

[25] Aslan F., Sahinoglu-Keskek N. (2022). The effect of home education on myopia progression in children during the COVID-19 pandemic. Eye.

[26] Liu J., Li B., Sun Y., Chen Q., Dang J. (2021). Adolescent Vision Health During the Outbreak of COVID-19: Association Between Digital Screen Use and Myopia Progression. Front. Pediatr..

[27] Wong C.W., Tsai A., Jonas J.B., Ohno-Matsui K., Chen J., Ang M., Ting D.S.W. (2021). Digital Screen Time During the COVID-19 Pandemic: Risk for a Further Myopia Boom?. Am. J. Ophthalmol..

[28] Ozturk Eyimaya A., Yalçin Irmak A. (2021). Relationship Between Parenting Practices and Children’s Screen Time During the COVID-19 Pandemic in Turkey. J. Pediatr. Nurs..

